# Noma: Time to Address a Collective Moral Failure

**DOI:** 10.4269/ajtmh.16-0997

**Published:** 2017-02-08

**Authors:** Raffaella Ravinetto

**Affiliations:** 1 Department of Public Health, Institute of Tropical Medicine, Antwerp, Belgium.

In this issue of the journal, Srour and others^1^ give a comprehensive overview of the history, epidemiology, etiology, pathogenesis, microbiology, prevention, diagnosis, and treatment of noma, a devastating orofacial gangrene that affects malnourished children in tropical regions. Even if exclusively present in tropical regions, noma is better described as a “poverty disease” rather than as a “tropical disease,” because it has accompanied extreme poverty and poor nutrition for centuries.^2^,^3^ With the exception of cases occurred in concentration camps during World War II,^2^,^4^ noma disappeared from Europe and North America by the end of the nineteenth century, thanks to the economic development and improved access to nutrition and health care.^1^,^3^ Today, it is particularly present in the sub-Saharan Africa “noma belt,” stretching from Senegal to Ethiopia.

The victims of noma are so neglected that their deaths are not included in mortality statistics^5^ or in the Global Burden of Diseases.^6^ Noma incidence is estimated to 30,000–140,000 cases, and its mortality at 85%. In addition, the disease, which is named after a Greek word (νoμη) meaning “devour” and indicating a process that develops very rapidly,^2^,^3^ leaves survivors with devastating sequelae: severe facial disfigurement and functional impairment hinder interpersonal relationships and trigger stigma and rejection from societal life.^1^,^3^,^4^,^7^ The pharmacological treatment is empirical and has not been tested in clinical trials.^5^ The surgical treatment of sequelae requires tertiary health care that is often unavailable, and it is estimated that at least 770,000 noma survivors remain in need of reconstructive surgery.^2^

Failure to provide adequate health care to people in need corresponds to failure to address the right to health, which is recognized in several human rights international instruments such as the Universal Declaration of Human Rights (article 25.1), the International Covenant on Economic, Social and Cultural Rights (article 12), and the Convention on the Rights of the Child (article 24.1). But the realization of the right to health care is a complex undertaking, closely related to the realization of other human rights, and linked to determinants of health.^8^ In the particular case of noma, the collective failure to acknowledge and address the human suffering that it causes should be seen as evidence of multiple rights violations, for example, the right to health care, food, water and sanitation, and adequate housing.^6^ Children affected by noma have even been cited in the resolution 19/7 on “The right to food” adopted by the United Nations Human Rights Council in 2012, as the “prime example of a violation” of the human right to food.

Today's strong call of Srour and others to put an end to the unacceptable neglect where noma patients, noma survivors, and their families are left, and in particular their advocacy for inclusion of noma in the World Health Organization (WHO) list of Neglected Tropical Diseases (NTDs), is neither exaggerated nor unrealistic. Noma patients and survivors desperately need national and international support,^6^ and the WHO Strategic and Technical Advisory Group for NTDs explicitly acknowledged that other conditions that constitute important health issues in populations affected by poverty could be classified as NTDs “for the purpose of advocacy to motivate action or research for the development of new solutions in low-resource settings”.^9^ The recent (May 28, 2016) inclusion in the NTDs list of mycetoma, a destructive inflammatory skin disease that affects the lower limbs in young adults with an important associated stigma, represents an encouraging precedent.^10^ The classification of noma as NTD could contribute to end the neglect of this devastating childhood disease, to mobilize resources for research on epidemiology, microbiology, and pathogenesis, and to facilitate the access to funding mechanisms, such as the European Developing Countries Clinical Trials Partnership (www.edctp.org), for targeted clinical research on prevention, diagnosis, and treatment. Because noma has multifactorial causes, the fight against noma should be framed in the strengthening of health systems in endemic countries and in their orientation to universal health coverage, that is, for ensuring that everyone—and especially the poorest and most marginalized populations—can obtain essential health services at high quality without suffering financial hardship.^11^

It has been long acknowledged that there is an ethical and human right imperative to address neglected diseases and populations.^12^ The call for the rights of children with noma should not be ignored anymore: it is time to mobilize energies and to allocate resources to care and cure the most vulnerable.
